# Ventilator-Associated Pneumonia in Paediatric Intensive Care Unit Patients: Microbiological Profile, Risk Factors, and Outcome

**DOI:** 10.7759/cureus.38189

**Published:** 2023-04-27

**Authors:** Piyali Bhattacharya, Arvind Kumar, Sanat Kumar Ghosh, Sudesh Kumar

**Affiliations:** 1 Paediatrics, Mata Gujri Memorial (MGM) Medical College, Kishanganj, IND; 2 Paediatrics, Patna Medical College, Patna, IND; 3 Paediatrics, Sarat Chandra Chattopadhyay Government Medical College & Hospital, Howrah, IND

**Keywords:** klebsiella, picu, ventilator associated pneumonia, mechanical ventilation, children

## Abstract

Introduction

Ventilator-associated pneumonia (VAP) is one of the dreaded events in sick children who are ventilated in the paediatric intensive care unit (PICU) and has a high mortality rate. So, there is a need to know the causative organisms, risk factors, and possible predictors in a particular PICU for prevention, early identification, and treatment to decrease morbidity and mortality. This study was planned with the objectives to determine the microbiological profile, associated risk factors, and outcome of VAP in children.

Methods

In this observational cross-sectional study conducted at Dr. B C Roy Post Graduate Institute of Paediatric Science, Kolkata, India, 37 VAP cases were diagnosed using clinical pulmonary infection score >6 and confirmed by tracheal culture and X-ray.

Results

The number of paediatric patients suffering from VAP was 37 (36.2%). The commonest age group involvement was one to five years. The microbiological profile included *Pseudomonas aeruginosa* (29.8%) and *Klebsiella* *pneumoniae* (21.6%) as the commonest organisms followed by *Staphylococcus aureus* (18.9%) and *Acinetobacter* (13.5%). The factors significantly associated with the increased frequency of VAP were the use of steroids, sedation, and reintubation. The mean duration of mechanical ventilation (MV) in VAP was 15 days compared to non-VAP (seven days), and the longer duration of ventilation was significantly associated with VAP (p=0.00001). Mortality in VAP was 48.54% compared to non-VAP (55.84%) with no significant association (p=0.0843) of VAP with death occurrence.

Conclusion

The present study showed that VAP occurrence is associated with prolonged duration of MV, PICU stay, and hospital stay but is not significantly associated with mortality. It also indicated that gram-negative bacteria were the most common VAP causative organisms in this cohort.

## Introduction

Mechanical ventilation (MV) is frequently required in managing critically ill children in an intensive care setting. However, it has its complications, such as the chance of developing ventilator-associated pneumonia (VAP) [1], which is the second-most common nosocomial infection after urinary tract infections in a paediatric intensive care unit (PICU), accounting for 20% of the cases [2]. Ventilator-associated pneumonia is different from community-acquired pneumonia in etiology, pathophysiology, risk factors, management strategies, and outcome [3]. It is defined as nosocomial pneumonia developing in mechanically ventilated patients after more than 48 hours of MV [3]. The reported mortality rate for patients with VAP is high and varies from 33% to 71% [4,5]. To prevent, identify and treat this condition, it is necessary to know the causative organisms and risk factors of VAP in a particular PICU, though these may vary across PICUs [6]. To that end, this study aimed to determine the microbiological profile, associated risk factors, and comparative outcome of VAP in children admitted to a PICU.

## Materials and methods

This prospective observational cross-sectional study was conducted in the PICU of Dr. B C Roy Post Graduate Institute of Paediatric Sciences, Kolkata, India, from September 2017 to February 2019. The participants were enrolled, observed and their data analyzed. Ethical approval (no. BCH/ME/PR/2658A) was received from the Institutional Ethics Committee, and written consent was obtained from patients. 

Study participants

All patients on invasive MV for more than 48 hours in the PICU of this institute were included in the study. They were closely examined and monitored for any features suggestive of VAP. All of these patients underwent septic screening, such as complete blood count (CBC), differential count (DC), C-reactive protein (CRP), blood and urine culture, tracheal aspirate and endotracheal tube tip culture; chest X-ray was also performed. The clinical pulmonary infection scoring (CPIS) system was used to diagnose VAP (Table 1). According to the criteria, CPIS score >6 would suggest VAP [7,8].

**Table 1:** Clinical pulmonary infection score (CPIS) ARDS: Acute respiratory distress syndrome, PaO2: Partial pressure of oxygen, FiO2: Fraction of inspired oxygen

CPIS points	0	1	2
Tracheal secretions	Rare	Abundant	Purulent
Leucocyte count (mm^3^)	>4000 and <11000	<4000 and > 11000	<4000 or >110009 +band forms
Temperature (c)	>36.5 and <38.4	>38.5 and <38.9	>39 or <36
PaO_2_/FiO_2 _(mmHg)	>240 or ARDS	-	=240 and no ARDS
Chest radiograph	No infiltrate	Diffuse infiltrate	Localized infiltrate
Culture of tracheal aspirate	Negative	-	Positive

The study excluded patients who were unwilling to give informed consent, less than one-month-old and more than 12 years of age, ventilated before admission to the PICU, had documented pneumonia at the time of PICU admission, or developed pneumonia within the first 48 hours of MV.

Procedure

All patients were ventilated through an endotracheal tube, which was changed only if it was blocked or displaced. A disposable ventilator circuit with a heated humidification system was used. An open method was used for suctioning secretions, the frequency of which depends on the amount of secretion. Patients were ventilated in a supine position with six-hourly changes to right lateral and left lateral decubitus positions. Furthermore, a nasogastric tube was inserted in all patients. No topical oropharyngeal antibiotic prophylaxis or selective digestive tract decontamination was done in any of the patients [6,9]. The endotracheal aspirate for microbiological culture was obtained using standard aseptic methods. Endotracheal secretions were collected by instilling 5 ml to 10 ml of sterile normal saline through an infant feeding tube inserted 30 cm into the endotracheal tubes. One end of the mucous extractor was connected to the infant feeding tube and the other end to an open suction pump [6]. The specimen collected was immediately transported aseptically to the laboratory. Tips of the endotracheal tubes were also sent for culture at the time of extubation or whenever the tube needed to be changed.

Operational definition

Data were collected by trained postgraduate trainees on pretested and predesigned proforma. The case was followed till discharge/leave against medical advice (LAMA), or the unfortunate event of death. The following relevant data were collected: temperature, recording of the partial pressure of oxygen (PaO2)/fraction of inspired oxygen (FiO2) ratio (mmHg), complete blood count (CBC), chest X-ray, and arterial blood gas (ABG) analysis. At the time of data collection, the VAP prevention protocol in the PICU included 30º elevation of the head of the bed, interventions to prevent peptic ulcer disease, and standardized oral care [10]. The VAP cases were managed initially by administering broad-spectrum empirical antibiotic therapy, followed by antibiotics suggested by the sensitivity pattern report.

Sampling method and sample size

Samples were taken using a simple random sampling technique. The sample size was calculated assuming a 95% confidence interval (CI), a 5% alpha error, a 20% prevalence of VAP, and a precision of 8% using Epi Info (CDC, Division of Health Informatics & Surveillance (DHIS), Atlanta, GA, USA) for a descriptive cross-sectional study. The total sample size came to 118.

Statistical analysis

We used the SPSS (IBM Corp., Armonk, NY, USA) software for statistical analysis. Data were entered into an Excel file (Microsoft Corp., Redmond, WA, USA). Categorical variables were expressed in count (%). All continuous variables were summarised using the mean (standard deviation (SD)) or median (interquartile range (IQR)). The categorical variable was measured using the chi-square test, and the risk estimate was performed by calculating the odds ratio and 95% CI. Continuous data were analyzed by t-test. A p-value less than 0.05 was considered significant.

## Results

In total, there were 603 patients admitted to the PICU during the study period, out of which 316 were mechanically ventilated; 214 cases were ruled out following the exclusion criteria, and the 102 remaining cases were included in the study. Diagnoses of VAP according to the CPIS criteria were made in 37 cases. Out of the 37 VAP cases, 11 (29.8%) were caused by *Pseudomonas aeruginosa*, and eight (21.6%) were caused by *Klebsiella*, while *Staphylococcus aureus *and* Acinetobacter* were responsible for seven (18.9%) and five (13.5%) cases, respectively (Table 2).

**Table 2:** Distribution of causative organisms in VAP cases VAP: Ventilator-associated pneumonia

Organism	Frequency	Percentage
Pseudomonas	11	29.80%
*Klebsiella*	8	21.60%
Staphylococcus aureus	7	18.90%
*Acinetobacter*	5	13.50%
Enterococcus	3	8.10%
Haemophilus influenzae	2	5.40%
Pneumococcus	1	2.70%
Total	37	100%

Though most cases belonged to the age group of one to five years (48.6%, p-value=0.314, 95% CI=13.0%, -7.73, 33.73), there was no age or sex preponderance in VAP cases (Table 3). A paediatric risk of mortality (PRISM) score >11.5 was present in 18 (48.648%) of the VAP cases (p=0.496, 95% CI=-22.68, 28.28), indicating that the PRISM score has no positive correlation with VAP occurrence.

**Table 3:** Risk factors and outcome of VAP VAP: Ventilator-associated pneumonia, PRISM: Paediatric risk of mortality score, H2: Histamine type 2, PPI: Proton pump inhibitor, MV: Mechanical ventilation, PICU: Paediatric intensive care unit

Risk factor	Frequency in VAP cases (%)	p-value	95% CI
Age			
<1 year	10, 27	0.314	7.07% (-15.12, 29.26)
1 to 5 years	18, 48.6	0.314	13.0% (-7.73, 33.73)
>5 years	9, 24.4	0.323	5.93% (-12.94, 24.80)
Gender			
Male	22, 59.45	0.321	6.70% (-14.98-28.38)
Female	15, 40.55	0.321	6.70% (-14.98-28.38)
PRISM score >11.5	18, 48.648	0.496	-22.68, 28.28
Use of steroids	18/37, 48.6	0.007	3.1579 (1.3294-7.5014)
Reintubation	15/37, 40.5	0.002245	4.2424 (1.6205-11.1063)
Use of neuromuscular blocking agent	7/37, 19	0.090	2.8 (0.8197-9.565)
Use of sedative	23/37, 62.1	0.0312	2.4643 (1.0753-5.6472)
Use of H2 blockers/PPI	35/37, 94.6	0.660	1.4583 (0.2685-7.9193)
Duration of MV (9 to 73 days)		<0.0001	0.0391 (0.0127-0.1199)
Duration of PICU stay (12 to 132 days)		<0.0001	0.0187 (0.0040-0.0864)
Duration of hospital stay (13 to 152 days)		0.0001	0.1534 (0.0615-0.3824)
Death	15, 40.54	0.5235	0.4355 to 2.2723

Steroids and sedatives were used in 18 (48.6%) and 23 (62.1%) VAP cases, respectively. On bivariate analysis, both steroid (p=.007, 95% CI=3.1579, 1.3294-7.5014) and sedative use (p=0.032145, 95% CI2.4643, 1.0753-5.6472) emerged as significant risk factors for VAP development. Reintubation was performed in a significant number (15 i.e., 40.5%) of VAP cases (p=0.002245, 95% CI=4.2424, 1.6205-11.1063) (Table 2). On the other hand, analysis of factors such as the use of neuromuscular blocking agents (p=0.090, 95% CI=2.8, 0.8197-9.565) and histamine type 2 (H2) blockers/proton pump inhibitor (PP)I (p=0.660, 95% CI=1.4583, 0.2685-7.9193) did not reveal any relation with the development of VAP (Table 3).

The mean duration of MV in VAP and non-VAP cases are 18.729 ± 16.208 days and 7.692 ± 3.844 days, respectively. The median duration of VAP cases is 15 days and seven days in non-VAP cases. The longer duration of MV was significantly associated with the development of VAP (p<0.0001, 95% CI=0.0391, 0.0127-0.1199) as seen in Table 3.

The mean duration of PICU stay of VAP and non-VAP cases were 24.4324 ± 27.3023 and 10.7692 ± 4.4008 days, respectively. The median duration of PICU stay for VAP cases is 17 days and 10 days for non-VAP cases. Evidently, the development of VAP was associated with significantly prolonged PICU stay (p<0.0001, 95% CI=0.0187, 0.0040-0.0864) as shown in Table 3.

The study shows that the majority of VAP cases (16, 43.24%) were hospitalized for more than 18 days. The maximum number of non-VAP cases (28, 40%) were hospitalized for ≤ 13 days. The mean duration of hospital stay of VAP and non-VAP cases were 28.4324 ± 27.3023 and 15.7692 ± 7.0860 days, respectively. The median duration in the cases of VAP is 20 days and 15 days for non-VAP cases. This study indicates that longer duration of hospital stay was significantly present in VAP cases (p=0.0001, 95% CI=0.1534, 0.0615-0.3824) (Table 3).

Fifteen (40.54%) of the VAP patients died, while 27 (41.53%) of the non-VAP patients died. The result indicates that mortality was not significantly associated with VAP development (p=0.5235, 95% CI=0.4355-2.2723) (Table 3).

## Discussion

To assess the microbiological profile, risk factors, and outcome of VAP in paediatric patients, 102 mechanically ventilated children admitted to the PICU of a tertiary care hospital were analyzed in this study. The frequency of VAP was measured at 36.27%. In this cohort, *Pseudomonas* emerged as the most common causative organism. The use of steroid and sedatives were documented as risk factors for VAP by bivariate analysis. Reintubation and longer duration of MV also emerged as risk factors for the same. The occurrence of VAP was found to result in a longer duration of PICU or hospital stay.

According to the microbiological profile, *Pseudomonas *(29.8%) and *Klebsiella* (21.6%) were the most common causative organisms of VAP, followed by *S. aureus* (18.9%) and *Acinetobacter* (13.5%). In the study by Foglia et al. [1], *P. aeruginosa *and* S. aureus* were the most commonly isolated organisms. Sharma et al. [11] revealed the microbiological profile of VAP to be *Pseudomonas *(31.48%), *S. aureus* (22%), and* Klebsiella* (14.8%). In some other studies like Chiru et al. [12] (*Pseudomonas* 57.7%, *Klebsiella* 17.7%), the profile differs given the different methods of specimen collection and different microbiological inhabitation and sensitivity patterns specific to each PICU setup. However, more or less unanimously in all the studies [1,13], including the present paper, gram-negative organisms were found to be the overwhelming majority in causing VAP.

The highest frequency of VAP cases was observed in the age group of one to five years (48.6%). Evidently, no particular age group is significantly associated with VAP (p=0.4089), similar to the study by Vedavathy et al. (p=0.269) and Kusahara et al. (p=0.267) [13,14]. Even though the proportion of males in VAP cases (22, 59.45%) is higher, the male gender is not significantly associated with VAP (p=0.4989). This finding is also corroborated by Chiru et al. (p=0.23) and Vedavathy et al. (p = 0.269) [12,14].

The mean PRISM score of VAP cases is 11 and that of non-VAP cases is 13.06. In both groups, a higher PRISM score is associated with greater mortality (p=0.00001). In a study by Roeleveld et al. [15], a PRISM score >10 was shown to be significantly associated with VAP development, which is not a finding in the present study. The use of steroids and sedatives, as well as reintubation and longer duration of MV, were noted as risk factors.

In this study, steroids were used in a significant number of VAP cases (p=.007). This finding is echoed in the studies of Mary et al. (p=0.003) and Huang et al. (p=0.007) [16,17]. Further, Liu et al.'s meta-analysis indicates that steroids are a risk factor for VAP [18]. This finding is corroborated by Hamid et al. (p=0.003) and Srinivasan et al. (p=0.001) [19,20].

Reintubation was performed in a significant number of VAP cases (15, 40.5%) (p=0.002245). Similar results were obtained in studies by Gnanaguru et al. (p=0.024) and Hamid et al. (p=0.02) [21,19]. Liu et al.’s meta-analysis suggests that reintubation might be a risk factor for VAP probably due to aspiration of oropharyngeal secretions or gastrointestinal contents during intubation or the interval between extubation and re-intubation [18]. Vedavathy et al. observed that 76% of the patients who had three to four intubations developed VAP [14].

The use of neuromuscular blocking agents was not significantly associated with VAP (7, 19%) nor with non-VAP cases (5, 7.6%) (p=0.090). Similar results were obtained by Kusahara et al. (p=0.16) [13]. However, conflicting observations were made by Fayon et al. (p=0.002) [22]. This disparity may be due to the use of neuromuscular blockade in a much smaller number of patients in the present study to be statistically relevant.

The use of H2 blockers/PPI was not found to be significantly associated with VAP development (p=0.6604). Similar findings were reflected in the studies by Chiru et al. (p=0.3), Vedavathy et al. (p=0.09), and Liu et al. (p=0.21) [12,14,18]. Huang et al. discovered that the use of H2 blockers/PPI was a significant risk factor in univariate analysis (p=0.006), which ceased to be so in multivariate analysis (p=0.112) [17]. However, Gnanaguru et al. proved it to be a risk factor for VAP on bivariate analysis (p=0.027) and was also seen in the studies by Elward et al. and Principi et al. [21,3,23]. The usage of H2 blockers can alter the gastric pH, thereby facilitating organism multiplication which when aspirated, can lead to the occurrence of VAP. However, in the present study, cuffed tubes were often used, which might have prevented aspiration of gastric contents. Also, H2 blockers were used extensively in both VAP and non-VAP cases, thus failing to produce any significant statistical difference.

Overall, 15 VAP patients (40.54%) died, 19 (51.35%) were discharged, and three (8.1%) took LAMA. The results indicate that mortality was not significantly associated with cases of VAP than in non-VAP cases (p=0.0843). The compounding effect of the severity of sickness is excluded in the present study by the insignificant difference in the mean PRISM score between the VAP and non-VAP cases. A similar finding is revealed in the studies by Chiru et al. (p=0.69), Almuneef et al., Vedavathy et al., and Huang et al. [12,24,14,17].

## Conclusions

Ventilator-associated pneumonia was observed to be mostly caused by gram-negative organisms, particularly *Pseudomonas *in this cohort. While VAP did not influence mortality, it did prolong the duration of ventilation, intensive care, and hospital stay, which in turn increased morbidity. In future research, more consistent and precise approaches to paediatric VAP diagnosis are needed to better define the attributable morbidity and mortality, pathophysiology, and appropriate interventions to prevent this disease. Judicious use of ventilator support and early weaning will reduce the incidence of VAP. More large multicentre studies are required for further evaluation of VAP in the paediatric population and for determining the best therapeutic approach to VAP and VAP prevention.

## References

[1] Foglia E, Meier MD, Elward A (2007). Ventilator-associated pneumonia in neonatal and pediatric intensive care unit patients. Clin Microbiol Rev.

[2] Richards MJ, Edwards JR, Culver DH, Gaynes RP (2000). Nosocomial infections in combined medical-surgical intensive care units in the United States. Infect Control Hosp Epidemiol.

[3] Elward AM, Warren DK, Fraser VJ (2002). Ventilator-associated pneumonia in pediatric intensive care unit patients: risk factors and outcomes. Pediatrics.

[4] American Thoracic Society, Infectious Diseases Society of America (2005). Guidelines for the management of adults with hospital-acquired, ventilator-associated, and healthcare-associated pneumonia. Am J Respir Crit Care Med.

[5] Edwards JR, Peterson KD, Andrus ML (2007). National Healthcare Safety Network (NHSN) Report, data summary for 2006, issued June 2007. Am J Infect Control.

[6] Awasthi S, Tahazzul M, Ambast A, Govil YC, Jain A (2013). Longer duration of mechanical ventilation was found to be associated with ventilator-associated pneumonia in children aged 1 month to 12 years in India. J Clin Epidemiol.

[7] Pugin J, Auckenthaler R, Mili N, Janssens JP, Lew PD, Suter PM (1991). Diagnosis of ventilator-associated pneumonia by bacteriologic analysis of bronchoscopic and nonbronchoscopic "blind" bronchoalveolar lavage fluid. Am Rev Respir Dis.

[8] Singh N, Rogers P, Atwood CW, Wagener MM, Yu VL (2000). Short-course empiric antibiotic therapy for patients with pulmonary infiltrates in the intensive care unit. A proposed solution for indiscriminate antibiotic prescription. Am J Respir Crit Care Med.

[9] Balakrishnan G, Aitchison T, Hallworth D, Morton NS (1992). Prospective evaluation of the Paediatric Risk of Mortality (PRISM) score. Arch Dis Child.

[10] Cooper VB, Haut C (2013). Preventing ventilator-associated pneumonia in children: an evidence-based protocol. Crit Care Nurse.

[11] Roeleveld PP, Guijt D, Kuijper EJ, Hazekamp MG, de Wilde RB, de Jonge E (2011). Ventilator-associated pneumonia in children after cardiac surgery in The Netherlands. Intensive Care Med.

[12] Chiru D, Crăciun A, Ţepeneu NF, Şipoş C, Bizerea T, Grecu A (2013). Incidence, risk factors, and nosocomial germs for ventilator-associated pneumonia in children. J Pediatr.

[13] Kusahara DM, Peterlini MA, Pedreira ML (2012). Oral care with 0.12% chlorhexidine for the prevention of ventilator-associated pneumonia in critically ill children: randomised, controlled and double blind trial. Int J Nurs Stud.

[14] Vedavathy S (2016). Clinical study of ventilator associated pneumonia in a tertiary care centre. Int J Contemp Pediatr.

[15] Elward AM (2003). Pediatric ventilator-associated pneumonia. Pediatr Infect Dis J.

[16] Lozada MA (2013). A retrospective study on ventilator-associated pneumonia among pediatric intensive care unit patients admitted in a tertiary hospital in Cebu City, Philippines. Eur Respir J.

[17] Huang WY, Lee MS, Lee CH, Tsao LY, Chiu HY (2012). Risk factors and outcomes of ventilator-associated pneumonia in children without pneumonia on admission. J Pediatr Resp Dis.

[18] Liu B, Li SQ, Zhang SM (2013). Risk factors of ventilator-associated pneumonia in pediatric intensive care unit: a systematic review and meta-analysis. J Thorac Dis.

[19] Hamid MH, Malik MA, Masood J, Zia A, Ahmad TM (2012). Ventilator-associated pneumonia in children. J Coll Physicians Surg Pak.

[20] Srinivasan R, Asselin J, Gildengorin G, Wiener-Kronish J, Flori HR (2009). A prospective study of ventilator-associated pneumonia in children. Pediatrics.

[21] Vijay G, Mandal A, Sankar J, Kapil A, Lodha R, Kabra SK (2018). Ventilator-associated pneumonia in pediatric intensive care unit: incidence, risk factors and etiological agents. Indian J Pediatr.

[22] Fayon MJ, Tucci M, Lacroix J, Farrell CA, Gauthier M, Lafleur L, Nadeau D (1997). Nosocomial pneumonia and tracheitis in a pediatric intensive care unit: a prospective study. Am J Respir Crit Care Med.

[23] Principi N, Esposito S (2007). Ventilator-associated pneumonia (VAP) in pediatric intensive care units. Pediatr Infect Dis J.

[24] Almuneef M, Memish ZA, Balkhy HH, Alalem H, Abutaleb A (2004). Ventilator-associated pneumonia in a pediatric intensive care unit in Saudi Arabia: a 30-month prospective surveillance. Infect Control Hosp Epidemiol.

